# The Discovery of T Cell–B Cell Cooperation

**DOI:** 10.3389/fimmu.2014.00377

**Published:** 2014-10-24

**Authors:** N. Avrion Mitchison

**Affiliations:** ^1^University College London, London, UK

**Keywords:** T cell, B cell, antibody, T–B cell collaboration, adaptive immunity

Until the mid-twentieth century, immunology had been very much a matter of soluble antibodies and their effect on the antigens of bacteria and viruses. Then, in the wartime and post-war years, a new area opened, of cell-mediated immunity, driven initially by interest in the ubiquitous rejection of homografts in man and animals. Experimental tolerance was a key discovery, that introducing donor-type cells before the ability to reject homografts had developed could prevent the rejection. Hašek in Czechoslovakia made the discovery independently in 1953 and by Billingham, Brent, and Medawar in Britain in 1954.

Proof that rejection of homografts is immunological in nature came from the discovery by the Medawar group that skin grafts are rejected more rapidly if the host has already rejected previous grafts from the same donor. My contribution was to show that this accelerated reaction could be transferred from one inbred mouse to another by means of spleen cells (1), work that I later continued in the laboratory of George Snell at Bar Harbor, ME (2).

Returning to UK, and after a period in Edinburgh University, I joined the National Institute of Medical Research, where Medawar had become director. My experience in Edinburgh with chicken erythrocytes had taught me the value of radioactive labeling (3), so I sought to adapt this technology (fairly new at the time) to tracking serum antibody levels in the small blood samples available in mouse studies. Down the passage worked Rosalind Pitt-Rivers, discoverer of the thyroid hormone T3, a great friend. Jointly we designed NIP-CAP, a structure related to T3 that can (i) serve as a powerful hapten because of its nitro and hydroxyl groups, (ii) couple smoothly to proteins to form part of immunogenic molecule, and (iii) can be prepared in radioactive form at the iodine residue and thus be used to assay binding of NI^131^P-CAP to its antibody (4). Together these properties opened the way to an easy mouse serology; indeed for a while, it became so widely used that the *European Journal of Immunology* accepted its name as not requiring further explanation.

My work focused on an aspect of immunological memory, the carrier effect. An individual primed by injection of a hapten–protein conjugate makes a full secondary anti-hapten antibody response only to the same conjugate, but not to the same hapten conjugated to another protein. This finding suggested to us that two cells might be involved, one recognizing the hapten and the other the carrier protein. To explore this possibility, we devised a serology applicable in mice (5). The small samples of serum available were appropriately diluted and then incubated with 10^−8^ M NI^131^P-CAP; their immunoglobulin was then precipitated by addition of ammonium sulfate solution and centrifuged, carrying the bound radioactive hapten down with it. By this method, anti-NIP antibody could be detected down to a concentration of ~10^−9^ M, as available with adoptively transferred spleen cells. This transfer system could then be used to explore the carrier effect as defined above. The secondary response obtained from the transferred spleen cells was indeed much reduced (~1000-fold) when the cells were stimulated with the same hapten (NIP) attached to a different carrier protein such as bovine serum albumin, compared to stimulation with the NIP-chicken γ-globulin originally used to immunize the cell donor. Importantly, the transferred anti-NIP response could be inhibited by injecting an excess of carrier protein, indicating that the carrier protein was itself recognized independently of the hapten that it carried, and thus that a second population of reactive cells was involved independent of those that recognized the hapten.

Our experimental design took spleen cells from mice immunized with NIP-OA (NIP conjugated with ovalbumin) plus adjuvant and transferred them into irradiated host mice that were then challenged with either NIP-BSA (NIP conjugated to bovine serum albumin) or NIP-OA (NIP conjugated to ovalbumin), both without adjuvant. The molar concentration anti-NIP antibody made in response was then measured, and its level titrated against the quantity of antigen in the challenge. Typically, mice needed a higher dose of the heterologous antigen (NIP-BSA) than of the homologous one (NIP-OA) to achieve the same level of anti-NIP antibody. Adding spleen cells from mice immunized with BSA alone to the transferred cell population increased sensitivity to NIP-BSA 10–100-fold, a finding that defines the “carrier effect.” The effect is specific, as the increase was not obtained with spleen cells from mice immunized with HSA (human serum albumin). These BSA-primed cells did not contribute directly to the anti-NIP antibody, as judged by allotype markers on the antibody; they acted only as “helper cells.”

Thus, these findings reveal a carrier effect mediated by the immune system, but not by antibody. To test for a T cell-mediated effect, cells were obtained from the spleen of mice that 7 days earlier had been irradiated and then reconstituted intravenously with 90 × 10^6^ syngeneic thymus cells and immunized with BSA, alum, and pertussis (6). These cells were tested for helper activity by transfer into irradiated syngeneic hosts, along with the usual NIP-BSA as immunogen. The transfer significantly increased the host anti-NIP antibody response, in proportion to the number of BSA-primed cells transferred.

Such experiments became easier later, when T cells could be manipulated by means of anti-theta antibody [reviewed by Raff (7, 8)]. By then, it had become clear that cooperation between T and B cells as revealed by the 1971 study considered here, most likely works through an antigen bridge between epitope-specific receptors on both cells. B cells, with their immunoglobulin receptors, recognizing the matrix of epitopes presented on the surface of T cells, became and remain the accepted mechanism of T–B cooperation in the immune response.

T cells and their interactions with other cells have become a major theme in immunology. Th interactions lie at the heart of inflammation and other aspects of immunological and infectious disease, and are increasingly being manipulated via monoclonal antibodies directed at cell surface markers and via cytokines. Advances in the molecular biology of the cell underpin these developments. These are extremely active fields, with much to offer in molecular cell biology and via therapeutic intervention.

## Conflict of Interest Statement

The author declares that the research was conducted in the absence of any commercial or financial relationships that could be construed as a potential conflict of interest.
